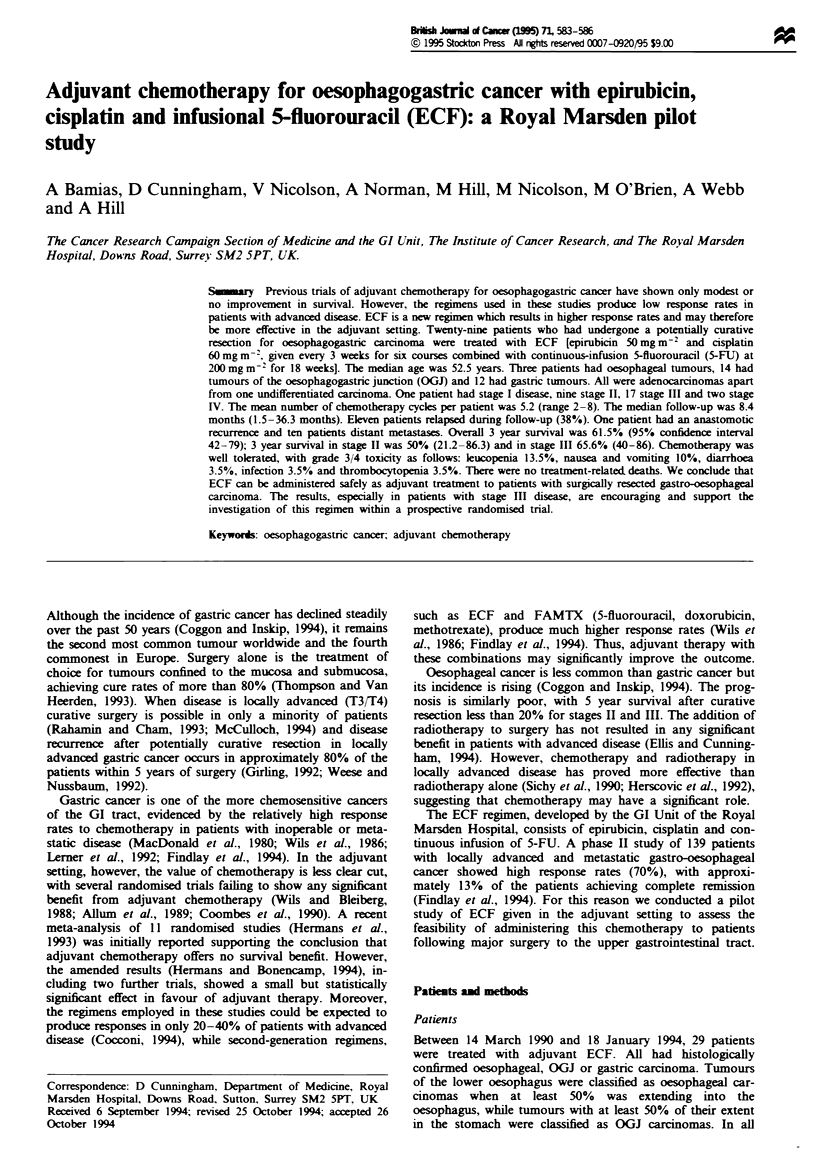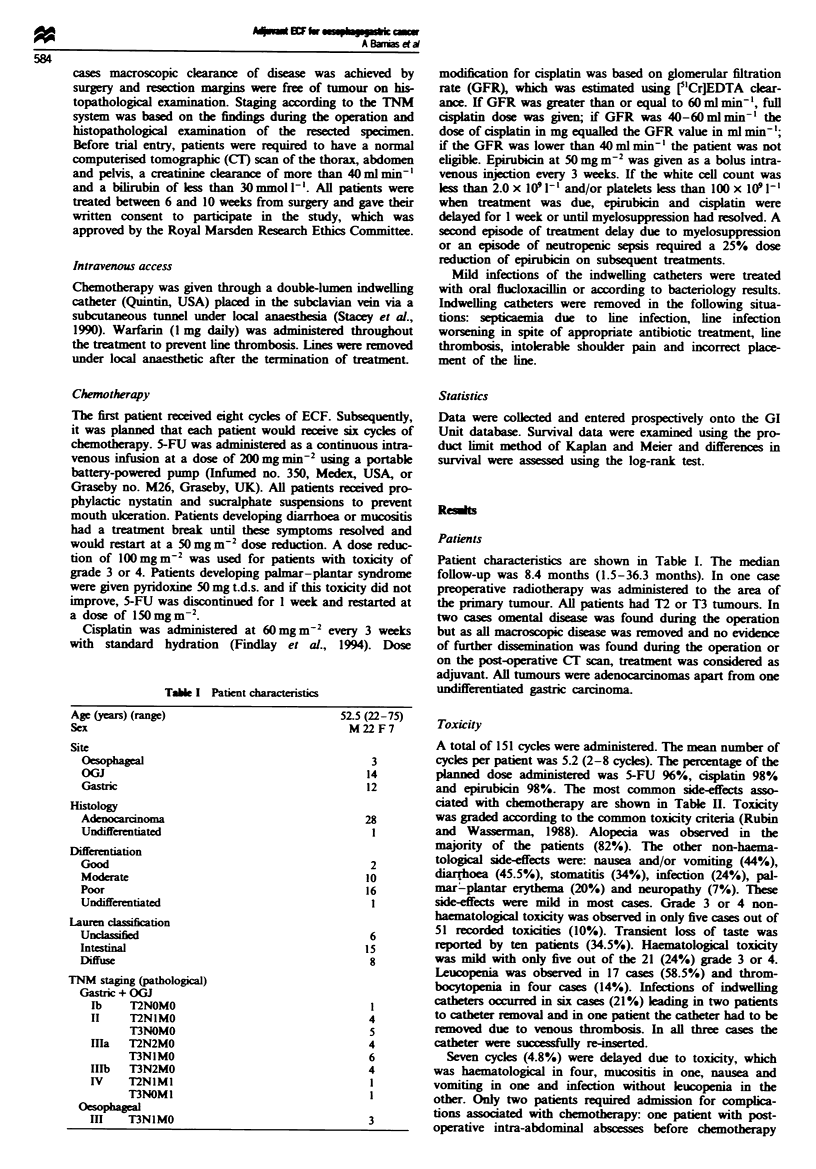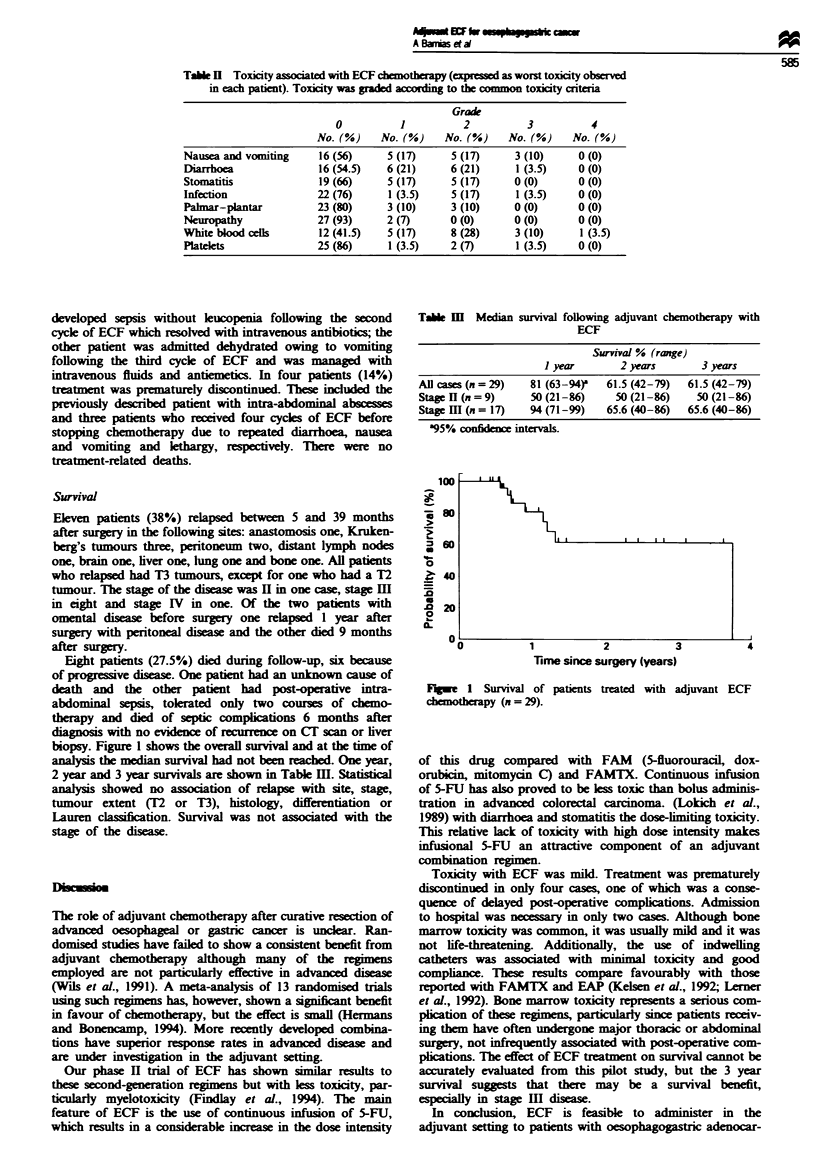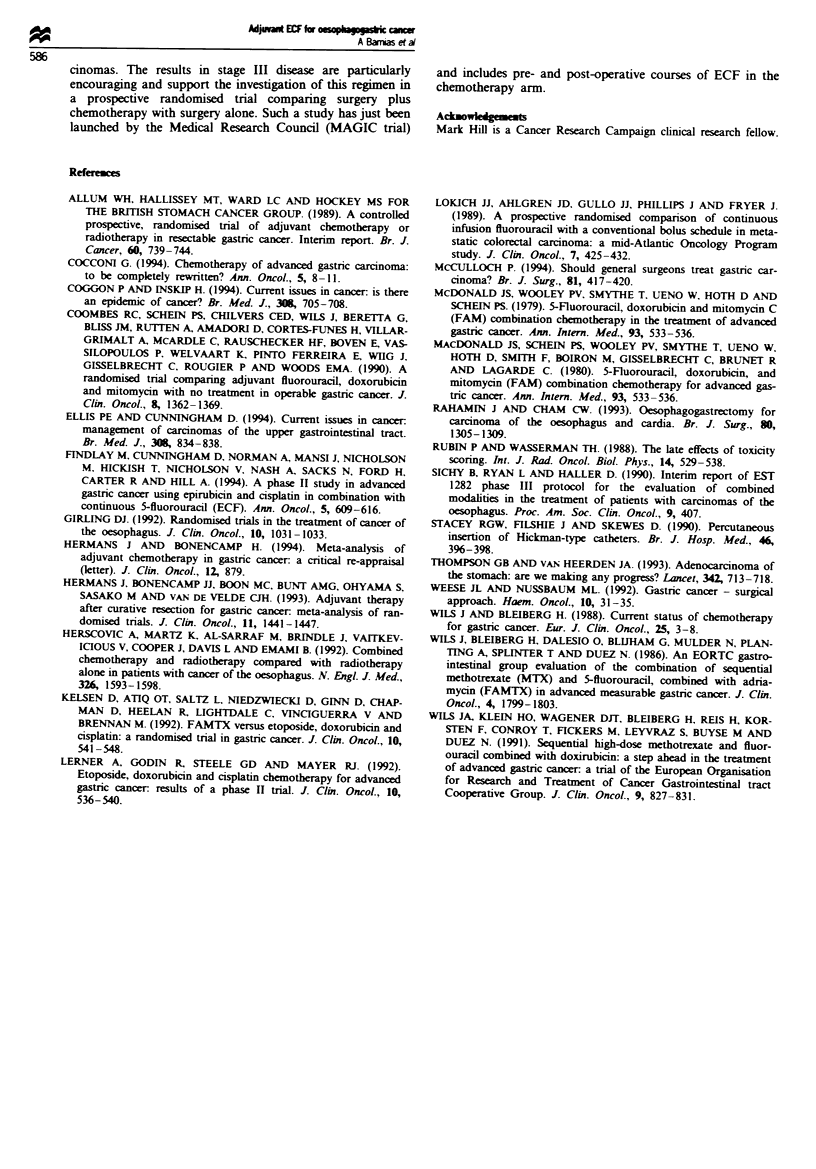# Adjuvant chemotherapy for oesophagogastric cancer with epirubicin, cisplatin and infusional 5-fluorouracil (ECF): a Royal Marsden pilot study.

**DOI:** 10.1038/bjc.1995.113

**Published:** 1995-03

**Authors:** A. Bamias, D. Cunningham, V. Nicolson, A. Norman, M. Hill, M. Nicolson, M. O'Brien, A. Webb, A. Hill

**Affiliations:** Cancer Research Campaign Section of Medicine, Institute of Cancer Research, Surrey, UK.

## Abstract

Previous trials of adjuvant chemotherapy for oesophagogastric cancer have shown only modest or no improvement in survival. However, the regimens used in these studies produce low response rates in patients with advanced disease. ECF is a new regimen which results in higher response rates and may therefore be more effective in the adjuvant setting. Twenty-nine patients who had undergone a potentially curative resection for oesphagogastric carcinoma were treated with ECF [epirubicin 50 mg m-2 and cisplatin 60 mg m-2 for 18 weeks]. The median age was 52.5 years. Three patients had oesophageal tumours, 14 had tumours of the oesophagogastric junction (OGJ) and 12 had gastric tumours. All were adenocarcinomas apart from one undifferentiated carcinoma. One patient had stage I disease, nine stage II, 17 stage II and two stage IV. The mean number of chemotherapy cycles per patient was 5.2 (range 2-8). The median follow-up was 8.4 months (1.5-36.3 months). Eleven patients relapsed during follow-up (38%). One patient had an anastomotic recurrence and ten patients distant metastases. Overall 3 year survival was 61.5% (95% confidence interval 42-79); 3 year survival in stage II was 50% (21.2-86.3) and in stage III 65.6% (40-86). Chemotherapy was well tolerated, with grade 3/4 toxicity as follows: leucopenia 13.5%, nausea and vomiting 10%, diarrhoea 3.5%, infection 3.5% and thrombocytopenia 3.5%. There were no treatment-related deaths. We conclude that ECF can be administered safely as adjuvant treatment to patients with surgically resected gastro-oesophageal carcinoma. The results, especially in patients with stage III disease, are encouraging and support the investigation of this regimen within a prospective randomised trial.


British Journal of Cancer (1995) 71, 583-586

© 1995 Stockton Press All rights reserved 0007-0920/95 $9.00

### Adjuvant chemotherapy for oesophagogastric cancer with epirubicin, cisplatin and infusional 5-fluorouracil (ECF): a Royal Marsden pilot study

A Bamias, D Cunningham, V Nicolson, A Norman, M Hill, M Nicolson, M O'Brien, A Webb and A Hill

*The Cancer Research Campaign Section of Medicine and the GI Unit, The Institute of Cancer Research, and The Royal Marsden Hospital, Downs Road, Surrey SM2 5PT, UK.*

**Summary** Previous trials of adjuvant chemotherapy for oesophagogastric cancer have shown only modest or no improvement in survival. However, the regimens used in these studies produce low response rates in patients with advanced disease. ECF is a new regimen which results in higher response rates and may therefore be more effective in the adjuvant setting. Twenty-nine patients who had undergone a potentially curative resection for oesophagogastric carcinoma were treated with ECF [epirubicin 50 mg m$^{-2}$ and cisplatin 60 mg m$^{-2}$, given every 3 weeks for six courses combined with continuous-infusion 5-fluorouracil (5-FU) at 200 mg m$^{-2}$ for 18 weeks]. The median age was 52.5 years. Three patients had oesophageal tumours, 14 had tumours of the oesophagogastric junction (OGJ) and 12 had gastric tumours. All were adenocarcinomas apart from one undifferentiated carcinoma. One patient had stage I disease, nine stage II, 17 stage III and two stage IV. The mean number of chemotherapy cycles per patient was 5.2 (range 2-8). The median follow-up was 8.4 months (1.5-36.3 months). Eleven patients relapsed during follow-up (38%). One patient had an anastomotic recurrence and ten patients distant metastases. Overall 3 year survival was 61.5% (95% confidence interval 42-79); 3 year survival in stage II was 50% (21.2-86.3) and in stage III 65.6% (40-86). Chemotherapy was well tolerated, with grade 3/4 toxicity as follows: leucopenia 13.5%, nausea and vomiting 10%, diarrhoea 3.5%, infection 3.5% and thrombocytopenia 3.5%. There were no treatment-related deaths. We conclude that ECF can be administered safely as adjuvant treatment to patients with surgically resected gastro-oesophageal carcinoma. The results, especially in patients with stage III disease, are encouraging and support the investigation of this regimen within a prospective randomised trial.

**Keywords**: oesophagogastric cancer; adjuvant chemotherapy

Although the incidence of gastric cancer has declined steadily over the past 50 years (Coggon and Inskip, 1994), it remains the second most common tumour worldwide and the fourth commonest in Europe. Surgery alone is the treatment of choice for tumours confined to the mucosa and submucosa, achieving cure rates of more than 80% (Thompson and Van Heerden, 1993). When disease is locally advanced (T3/T4) curative surgery is possible in only a minority of patients (Rahamin and Cham, 1993; McCulloch, 1994) and disease recurrence after potentially curative resection in locally advanced gastric cancer occurs in approximately 80% of the patients within 5 years of surgery (Girling, 1992; Weese and Nussbaum, 1992).

Gastric cancer is one of the more chemosensitive cancers of the GI tract, evidenced by the relatively high response rates to chemotherapy in patients with inoperable or metastatic disease (MacDonald *et al.*, 1980; Wils *et al.*, 1986; Lerner *et al.*, 1992; Findlay *et al.*, 1994). In the adjuvant setting, however, the value of chemotherapy is less clear cut, with several randomised trials failing to show any significant benefit from adjuvant chemotherapy (Wils and Bleiberg, 1988; Allum *et al.*, 1989; Coombes *et al.*, 1990). A recent meta-analysis of 11 randomised studies (Hermans *et al.*, 1993) was initially reported supporting the conclusion that adjuvant chemotherapy offers no survival benefit. However, the amended results (Hermans and Bonencamp, 1994), including two further trials, showed a small but statistically significant effect in favour of adjuvant therapy. Moreover, the regimens employed in these studies could be expected to produce responses in only 20-40% of patients with advanced disease (Cocconi, 1994), while second-generation regimens, such as ECF and FAMTX (5-fluorouracil, doxorubicin, methotrexate), produce much higher response rates (Wils *et al.*, 1986; Findlay *et al.*, 1994). Thus, adjuvant therapy with these combinations may significantly improve the outcome.

Oesophageal cancer is less common than gastric cancer but its incidence is rising (Coggon and Inskip, 1994). The prognosis is similarly poor, with 5 year survival after curative resection less than 20% for stages II and III. The addition of radiotherapy to surgery has not resulted in any significant benefit in patients with advanced disease (Ellis and Cunningham, 1994). However, chemotherapy and radiotherapy in locally advanced disease has proved more effective than radiotherapy alone (Sichy *et al.*, 1990; Herscovic *et al.*, 1992), suggesting that chemotherapy may have a significant role.

The ECF regimen, developed by the GI Unit of the Royal Marsden Hospital, consists of epirubicin, cisplatin and continuous infusion of 5-FU. A phase II study of 139 patients with locally advanced and metastatic gastro-oesophageal cancer showed high response rates (70%), with approximately 13% of the patients achieving complete remission (Findlay *et al.*, 1994). For this reason we conducted a pilot study of ECF given in the adjuvant setting to assess the feasibility of administering this chemotherapy to patients following major surgery to the upper gastrointestinal tract.

### Patients and methods

### *Patients*

Between 14 March 1990 and 18 January 1994, 29 patients were treated with adjuvant ECF. All had histologically confirmed oesophageal, OGJ or gastric carcinoma. Tumours of the lower oesophagus were classified as oesophageal carcinomas when at least 50% was extending into the oesophagus, while tumours with at least 50% of their extent in the stomach were classified as OGJ carcinomas. In all

Correspondence: D Cunningham, Department of Medicine, Royal Marsden Hospital, Downs Road, Sutton, Surrey SM2 5PT, UK
Received 6 September 1994; revised 25 October 1994; accepted 26 October 1994

cases macroscopic clearance of disease was achieved by surgery and resection margins were free of tumour on histopathological examination. Staging according to the TNM system was based on the findings during the operation and histopathological examination of the resected specimen. Before trial entry, patients were required to have a normal computerised tomographic (CT) scan of the thorax, abdomen and pelvis, a creatinine clearance of more than 40 ml $min^{-1}$ and a bilirubin of less than 30 mmol $l^{-1}$. All patients were treated between 6 and 10 weeks from surgery and gave their written consent to participate in the study, which was approved by the Royal Marsden Research Ethics Committee.

*Intravenous access*

Chemotherapy was given through a double-lumen indwelling catheter (Quintin, USA) placed in the subclavian vein via a subcutaneous tunnel under local anaesthesia (Stacey *et al.*, 1990). Warfarin (1 mg daily) was administered throughout the treatment to prevent line thrombosis. Lines were removed under local anaesthetic after the termination of treatment.

*Chemotherapy*

The first patient received eight cycles of ECF. Subsequently, it was planned that each patient would receive six cycles of chemotherapy. 5-FU was administered as a continuous intravenous infusion at a dose of 200 mg $min^{-2}$ using a portable battery-powered pump (Infumed no. 350, Medex, USA, or Graseby no. M26, Graseby, UK). All patients received prophylactic nystatin and sucralphate suspensions to prevent mouth ulceration. Patients developing diarrhoea or mucositis had a treatment break until these symptoms resolved and would restart at a 50 mg $m^{-2}$ dose reduction. A dose reduction of 100 mg $m^{-2}$ was used for patients with toxicity of grade 3 or 4. Patients developing palmar–plantar syndrome were given pyridoxine 50 mg t.d.s. and if this toxicity did not improve, 5-FU was discontinued for 1 week and restarted at a dose of 150 mg $m^{-2}$.

Cisplatin was administered at 60 mg $m^{-2}$ every 3 weeks with standard hydration (Findlay *et al.*, 1994). Dose modification for cisplatin was based on glomerular filtration rate (GFR), which was estimated using [$^{51}$Cr]EDTA clearance. If GFR was greater than or equal to 60 ml $min^{-1}$, full cisplatin dose was given; if GFR was 40–60 ml $min^{-1}$ the dose of cisplatin in mg equalled the GFR value in ml $min^{-1}$; if the GFR was lower than 40 ml $min^{-1}$ the patient was not eligible. Epirubicin at 50 mg $m^{-2}$ was given as a bolus intravenous injection every 3 weeks. If the white cell count was less than $2.0 \times 10^9$ $l^{-1}$ and/or platelets less than $100 \times 10^9$ $l^{-1}$ when treatment was due, epirubicin and cisplatin were delayed for 1 week or until myelosuppression had resolved. A second episode of treatment delay due to myelosuppression or an episode of neutropenic sepsis required a 25% dose reduction of epirubicin on subsequent treatments.

Mild infections of the indwelling catheters were treated with oral flucloxacillin or according to bacteriology results. Indwelling catheters were removed in the following situations: septicaemia due to line infection, line infection worsening in spite of appropriate antibiotic treatment, line thrombosis, intolerable shoulder pain and incorrect placement of the line.

**Table I** Patient characteristics

| | | |
|---|---|---|
| Age (years) (range) | | 52.5 (22–75) |
| Sex | | M 22 F 7 |
| Site | | |
| Oesophageal | | 3 |
| OGJ | | 14 |
| Gastric | | 12 |
| Histology | | |
| Adenocarcinoma | | 28 |
| Undifferentiated | | 1 |
| Differentiation | | |
| Good | | 2 |
| Moderate | | 10 |
| Poor | | 16 |
| Undifferentiated | | 1 |
| Lauren classification | | |
| Unclassified | | 6 |
| Intestinal | | 15 |
| Diffuse | | 8 |
| TNM staging (pathological) | | |
| Gastric + OGJ | | |
| Ib | T2N0M0 | 1 |
| II | T2N1M0 | 4 |
| | T3N0M0 | 5 |
| IIIa | T2N2M0 | 4 |
| | T3N1M0 | 6 |
| IIIb | T3N2M0 | 4 |
| IV | T2N1M1 | 1 |
| | T3N0M1 | 1 |
| Oesophageal | | |
| III | T3N1M0 | 3 |

*Statistics*

Data were collected and entered prospectively onto the GI Unit database. Survival data were examined using the product limit method of Kaplan and Meier and differences in survival were assessed using the log-rank test.

### Results

*Patients*

Patient characteristics are shown in Table I. The median follow-up was 8.4 months (1.5–36.3 months). In one case preoperative radiotherapy was administered to the area of the primary tumour. All patients had T2 or T3 tumours. In two cases omental disease was found during the operation but as all macroscopic disease was removed and no evidence of further dissemination was found during the operation or on the post-operative CT scan, treatment was considered as adjuvant. All tumours were adenocarcinomas apart from one undifferentiated gastric carcinoma.

*Toxicity*

A total of 151 cycles were administered. The mean number of cycles per patient was 5.2 (2–8 cycles). The percentage of the planned dose administered was 5-FU 96%, cisplatin 98% and epirubicin 98%. The most common side-effects associated with chemotherapy are shown in Table II. Toxicity was graded according to the common toxicity criteria (Rubin and Wasserman, 1988). Alopecia was observed in the majority of the patients (82%). The other non-haematological side-effects were: nausea and/or vomiting (44%), diarrhoea (45.5%), stomatitis (34%), infection (24%), palmar–plantar erythema (20%) and neuropathy (7%). These side-effects were mild in most cases. Grade 3 or 4 non-haematological toxicity was observed in only five cases out of 51 recorded toxicities (10%). Transient loss of taste was reported by ten patients (34.5%). Haematological toxicity was mild with only five out of the 21 (24%) grade 3 or 4. Leucopenia was observed in 17 cases (58.5%) and thrombocytopenia in four cases (14%). Infections of indwelling catheters occurred in six cases (21%) leading in two patients to catheter removal and in one patient the catheter had to be removed due to venous thrombosis. In all three cases the catheter were successfully re-inserted.

Seven cycles (4.8%) were delayed due to toxicity, which was haematological in four, mucositis in one, nausea and vomiting in one and infection without leucopenia in the other. Only two patients required admission for complications associated with chemotherapy: one patient with post-operative intra-abdominal abscesses before chemotherapy

**Table II** Toxicity associated with ECF chemotherapy (expressed as worst toxicity observed in each patient). Toxicity was graded according to the common toxicity criteria

| | Grade 0 No. (%) | Grade 1 No. (%) | Grade 2 No. (%) | Grade 3 No. (%) | Grade 4 No. (%) |
|---|---|---|---|---|---|
| Nausea and vomiting | 16 (56) | 5 (17) | 5 (17) | 3 (10) | 0 (0) |
| Diarrhoea | 16 (54.5) | 6 (21) | 6 (21) | 1 (3.5) | 0 (0) |
| Stomatitis | 19 (66) | 5 (17) | 5 (17) | 0 (0) | 0 (0) |
| Infection | 22 (76) | 1 (3.5) | 5 (17) | 1 (3.5) | 0 (0) |
| Palmar-plantar | 23 (80) | 3 (10) | 3 (10) | 0 (0) | 0 (0) |
| Neuropathy | 27 (93) | 2 (7) | 0 (0) | 0 (0) | 0 (0) |
| White blood cells | 12 (41.5) | 5 (17) | 8 (28) | 3 (10) | 1 (3.5) |
| Platelets | 25 (86) | 1 (3.5) | 2 (7) | 1 (3.5) | 0 (0) |

developed sepsis without leucopenia following the second cycle of ECF which resolved with intravenous antibiotics; the other patient was admitted dehydrated owing to vomiting following the third cycle of ECF and was managed with intravenous fluids and antiemetics. In four patients (14%) treatment was prematurely discontinued. These included the previously described patient with intra-abdominal abscesses and three patients who received four cycles of ECF before stopping chemotherapy due to repeated diarrhoea, nausea and vomiting and lethargy, respectively. There were no treatment-related deaths.

*Survival*

Eleven patients (38%) relapsed between 5 and 39 months after surgery in the following sites: anastomosis one, Krukenberg's tumours three, peritoneum two, distant lymph nodes one, brain one, liver one, lung one and bone one. All patients who relapsed had T3 tumours, except for one who had a T2 tumour. The stage of the disease was II in one case, stage III in eight and stage IV in one. Of the two patients with omental disease before surgery one relapsed 1 year after surgery with peritoneal disease and the other died 9 months after surgery.

Eight patients (27.5%) died during follow-up, six because of progressive disease. One patient had an unknown cause of death and the other patient had post-operative intra-abdominal sepsis, tolerated only two courses of chemotherapy and died of septic complications 6 months after diagnosis with no evidence of recurrence on CT scan or liver biopsy. Figure 1 shows the overall survival and at the time of analysis the median survival had not been reached. One year, 2 year and 3 year survivals are shown in Table III. Statistical analysis showed no association of relapse with site, stage, tumour extent (T2 or T3), histology, differentiation or Lauren classification. Survival was not associated with the stage of the disease.

**Table III** Median survival following adjuvant chemotherapy with ECF

| | Survival % (range) 1 year | 2 years | 3 years |
|---|---|---|---|
| All cases ($n = 29$) | 81 (63–94)[a] | 61.5 (42–79) | 61.5 (42–79) |
| Stage II ($n = 9$) | 50 (21–86) | 50 (21–86) | 50 (21–86) |
| Stage III ($n = 17$) | 94 (71–99) | 65.6 (40–86) | 65.6 (40–86) |

[a]95% confidence intervals.

**Figure 1** Survival of patients treated with adjuvant ECF chemotherapy ($n = 29$).

### Discussion

The role of adjuvant chemotherapy after curative resection of advanced oesophageal or gastric cancer is unclear. Randomised studies have failed to show a consistent benefit from adjuvant chemotherapy although many of the regimens employed are not particularly effective in advanced disease (Wils *et al.*, 1991). A meta-analysis of 13 randomised trials using such regimens has, however, shown a significant benefit in favour of chemotherapy, but the effect is small (Hermans and Bonencamp, 1994). More recently developed combinations have superior response rates in advanced disease and are under investigation in the adjuvant setting.

Our phase II trial of ECF has shown similar results to these second-generation regimens but with less toxicity, particularly myelotoxicity (Findlay *et al.*, 1994). The main feature of ECF is the use of continuous infusion of 5-FU, which results in a considerable increase in the dose intensity of this drug compared with FAM (5-fluorouracil, doxorubicin, mitomycin C) and FAMTX. Continuous infusion of 5-FU has also proved to be less toxic than bolus administration in advanced colorectal carcinoma. (Lokich *et al.*, 1989) with diarrhoea and stomatitis the dose-limiting toxicity. This relative lack of toxicity with high dose intensity makes infusional 5-FU an attractive component of an adjuvant combination regimen.

Toxicity with ECF was mild. Treatment was prematurely discontinued in only four cases, one of which was a consequence of delayed post-operative complications. Admission to hospital was necessary in only two cases. Although bone marrow toxicity was common, it was usually mild and it was not life-threatening. Additionally, the use of indwelling catheters was associated with minimal toxicity and good compliance. These results compare favourably with those reported with FAMTX and EAP (Kelsen *et al.*, 1992; Lerner *et al.*, 1992). Bone marrow toxicity represents a serious complication of these regimens, particularly since patients receiving them have often undergone major thoracic or abdominal surgery, not infrequently associated with post-operative complications. The effect of ECF treatment on survival cannot be accurately evaluated from this pilot study, but the 3 year survival suggests that there may be a survival benefit, especially in stage III disease.

In conclusion, ECF is feasible to administer in the adjuvant setting to patients with oesophagogastric adenocarcinomas. The results in stage III disease are particularly encouraging and support the investigation of this regimen in a prospective randomised trial comparing surgery plus chemotherapy with surgery alone. Such a study has just been launched by the Medical Research Council (MAGIC trial) and includes pre- and post-operative courses of ECF in the chemotherapy arm.

**Acknowledgements**

Mark Hill is a Cancer Research Campaign clinical research fellow.

**References**

ALLUM WH, HALLISSEY MT, WARD LC AND HOCKEY MS FOR THE BRITISH STOMACH CANCER GROUP. (1989). A controlled prospective, randomised trial of adjuvant chemotherapy or radiotherapy in resectable gastric cancer. Interim report. *Br. J. Cancer*, **60**, 739–744.

COCCONI G. (1994). Chemotherapy of advanced gastric carcinoma: to be completely rewritten? *Ann. Oncol.*, **5**, 8–11.

COGGON P AND INSKIP H. (1994). Current issues in cancer: is there an epidemic of cancer? *Br. Med. J.*, **308**, 705–708.

COOMBES RC, SCHEIN PS, CHILVERS CED, WILS J, BERETTA G, BLISS JM, RUTTEN A, AMADORI D, CORTES-FUNES H, VILLAR-GRIMALT A, MCARDLE C, RAUSCHECKER HF, BOVEN E, VASSILOPOULOS P, WELVAART K, PINTO FERREIRA E, WIIG J, GISSELBRECHT C, ROUGIER P AND WOODS EMA. (1990). A randomised trial comparing adjuvant fluorouracil, doxorubicin and mitomycin with no treatment in operable gastric cancer. *J. Clin. Oncol.*, **8**, 1362–1369.

ELLIS PE AND CUNNINGHAM D. (1994). Current issues in cancer: management of carcinomas of the upper gastrointestinal tract. *Br. Med. J.*, **308**, 834–838.

FINDLAY M, CUNNINGHAM D, NORMAN A, MANSI J, NICHOLSON M, HICKISH T, NICHOLSON V, NASH A, SACKS N, FORD H, CARTER R AND HILL A. (1994). A phase II study in advanced gastric cancer using epirubicin and cisplatin in combination with continuous 5-fluorouracil (ECF). *Ann. Oncol.*, **5**, 609–616.

GIRLING DJ. (1992). Randomised trials in the treatment of cancer of the oesophagus. *J. Clin. Oncol.*, **10**, 1031–1033.

HERMANS J AND BONENCAMP H. (1994). Meta-analysis of adjuvant chemotherapy in gastric cancer: a critical re-appraisal (letter). *J. Clin. Oncol.*, **12**, 879.

HERMANS J, BONENCAMP JJ, BOON MC, BUNT AMG, OHYAMA S, SASAKO M AND VAN DE VELDE CJH. (1993). Adjuvant therapy after curative resection for gastric cancer: meta-analysis of randomised trials. *J. Clin. Oncol.*, **11**, 1441–1447.

HERSCOVIC A, MARTZ K, AL-SARRAF M, BRINDLE J, VAITKEVICIOUS V, COOPER J, DAVIS L AND EMAMI B. (1992). Combined chemotherapy and radiotherapy compared with radiotherapy alone in patients with cancer of the oesophagus. *N. Engl. J. Med.*, **326**, 1593–1598.

KELSEN D, ATIQ OT, SALTZ L, NIEDZWIECKI D, GINN D, CHAPMAN D, HEELAN R, LIGHTDALE C, VINCIGUERRA V AND BRENNAN M. (1992). FAMTX versus etoposide, doxorubicin and cisplatin: a randomised trial in gastric cancer. *J. Clin. Oncol.*, **10**, 541–548.

LERNER A, GODIN R, STEELE GD AND MAYER RJ. (1992). Etoposide, doxorubicin and cisplatin chemotherapy for advanced gastric cancer: results of a phase II trial. *J. Clin. Oncol.*, **10**, 536–540.

LOKICH JJ, AHLGREN JD, GULLO JJ, PHILLIPS J AND FRYER J. (1989). A prospective randomised comparison of continuous infusion fluorouracil with a conventional bolus schedule in metastatic colorectal carcinoma: a mid-Atlantic Oncology Program study. *J. Clin. Oncol.*, **7**, 425–432.

MCCULLOCH P. (1994). Should general surgeons treat gastric carcinoma? *Br. J. Surg.*, **81**, 417–420.

MCDONALD JS, WOOLEY PV, SMYTHE T, UENO W, HOTH D AND SCHEIN PS. (1979). 5-Fluorouracil, doxorubicin and mitomycin C (FAM) combination chemotherapy in the treatment of advanced gastric cancer. *Ann. Intern. Med.*, **93**, 533–536.

MACDONALD JS, SCHEIN PS, WOOLEY PV, SMYTHE T, UENO W, HOTH D, SMITH F, BOIRON M, GISSELBRECHT C, BRUNET R AND LAGARDE C. (1980). 5-Fluorouracil, doxorubicin, and mitomycin (FAM) combination chemotherapy for advanced gastric cancer. *Ann. Intern. Med.*, **93**, 533–536.

RAHAMIN J AND CHAM CW. (1993). Oesophagogastrectomy for carcinoma of the oesophagus and cardia. *Br. J. Surg.*, **80**, 1305–1309.

RUBIN P AND WASSERMAN TH. (1988). The late effects of toxicity scoring. *Int. J. Rad. Oncol. Biol. Phys.*, **14**, 529–538.

SICHY B, RYAN L AND HALLER D. (1990). Interim report of EST 1282 phase III protocol for the evaluation of combined modalities in the treatment of patients with carcinomas of the oesophagus. *Proc. Am. Soc. Clin. Oncol.*, **9**, 407.

STACEY RGW, FILSHIE J AND SKEWES D. (1990). Percutaneous insertion of Hickman-type catheters. *Br. J. Hosp. Med.*, **46**, 396–398.

THOMPSON GB AND VAN HEERDEN JA. (1993). Adenocarcinoma of the stomach: are we making any progress? *Lancet*, **342**, 713–718.

WEESE JL AND NUSSBAUM ML. (1992). Gastric cancer – surgical approach. *Haem. Oncol.*, **10**, 31–35.

WILS J AND BLEIBERG H. (1988). Current status of chemotherapy for gastric cancer. *Eur. J. Clin. Oncol.*, **25**, 3–8.

WILS J, BLEIBERG H, DALESIO O, BLIJHAM G, MULDER N, PLANTING A, SPLINTER T AND DUEZ N. (1986). An EORTC gastrointestinal group evaluation of the combination of sequential methotrexate (MTX) and 5-fluorouracil, combined with adriamycin (FAMTX) in advanced measurable gastric cancer. *J. Clin. Oncol.*, **4**, 1799–1803.

WILS JA, KLEIN HO, WAGENER DJT, BLEIBERG H, REIS H, KORSTEN F, CONROY T, FICKERS M, LEYVRAZ S, BUYSE M AND DUEZ N. (1991). Sequential high-dose methotrexate and fluorouracil combined with doxirubicin: a step ahead in the treatment of advanced gastric cancer: a trial of the European Organisation for Research and Treatment of Cancer Gastrointestinal tract Cooperative Group. *J. Clin. Oncol.*, **9**, 827–831.